# Traumatic posterior urethral fistula to hip joint following gunshot injury: a case report

**DOI:** 10.1186/1752-1947-3-133

**Published:** 2009-11-18

**Authors:** Ahmad Rezaee, Behzad Narouie, Rahim Haji-Rajabi, Mohammad Ghasemi-rad, Abdolsamad Shikhzadeh

**Affiliations:** 1 Faculty of Medicine, Zahedan University of Medical Sciences, Zahedan, Iran; 2 Clinical Research Development Center, Ali-ebne-Abitaleb Hospital, Zahedan University of Medical Sciences, Zahedan, Iran; 3 Genius and Talented Student Organization, Student Research Committee (SRC), Urmia University of Medical Sciences, Urmia, Iran

## Abstract

**Introduction:**

Urinary system fistula to the hip joint is a rare complication. We report a case of delayed posterior urethral fistula to the hip joint following penetrating gunshot wound injury.

**Case presentation:**

A 37-year-old Iranian Balochi male was shot with a firearm in the superior part of his right pelvis. He underwent primary closure on the same day. Ten months later, he developed urinary retention. He underwent retrograde urethrography and antegrade cystography which showed a stricture measuring 5 cm in length. There was also a history of progressive pain in the right hip joint accompanied by low grade fever which started 2 months after the initial injury. Hip X-ray showed evidence of an acetabular cavity and femoral head destruction diagnostic of complicated septic arthritis. The patient subsequently underwent reconstructive surgery for the urethral stricture and urethral fistula via a transperineal approach followed by total hip arthroplasty.

**Conclusion:**

Hip joint contamination with urine following a urethro-acetabular fistula can lead to severe and disabling complications such as septic arthritis. We recommend that every clinician should keep these fistulas in mind as a complication of penetrating urethral injury and every attempt should be made for their early diagnosis and prompt treatment.

## Introduction

Urethral injuries are uncommon and occur most often in men. The membranous urethra which passes through the pelvic floor and voluntary urinary sphincter are the portion of posterior urethra most likely to be injured [1].

Blunt trauma of the posterior urethra accounts for 90% of urethral injuries while penetrating injuries are extremely rare [1]. The physical findings for penetrating urethral trauma are the same as those found in blunt urethral trauma, i.e. high riding prostate, blood at the urethral meatus, bladder distension, inability to void, gross hematuria, scrotal, perineal, or penile hematoma, and difficulty passing Foley's catheter [2]. The late complications of posterior urethral injury are bleeding, urinary extravasation, pelvic abscess, and destruction of the posterior urethra, urinary diversion, urethral fistulas and urethral stricture [2]. Peri-urethral or perivesical urinary extravasations seen on retrograde urethrography usually confirm the diagnosis [2]. Surgical reconstruction such as posterior urethroplasty via a perineal approach remains the cornerstone in management of urethral injuries, and if complications are avoided, the prognosis is excellent.

## Case presentation

**Figure 1:**
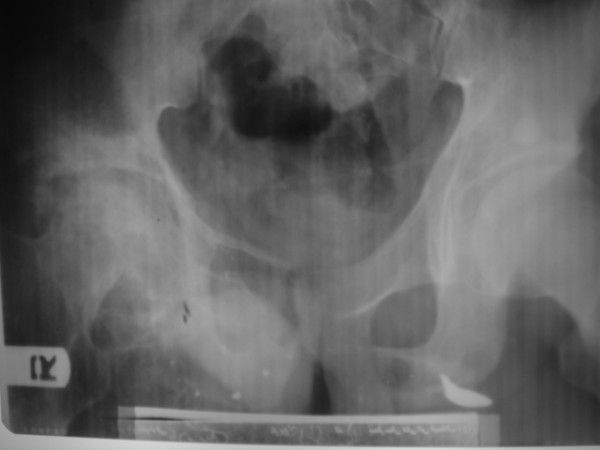
**Plain X-ray of the right hip joint showing destruction of the joint cavity and femoral head**.

**Figure 2:**
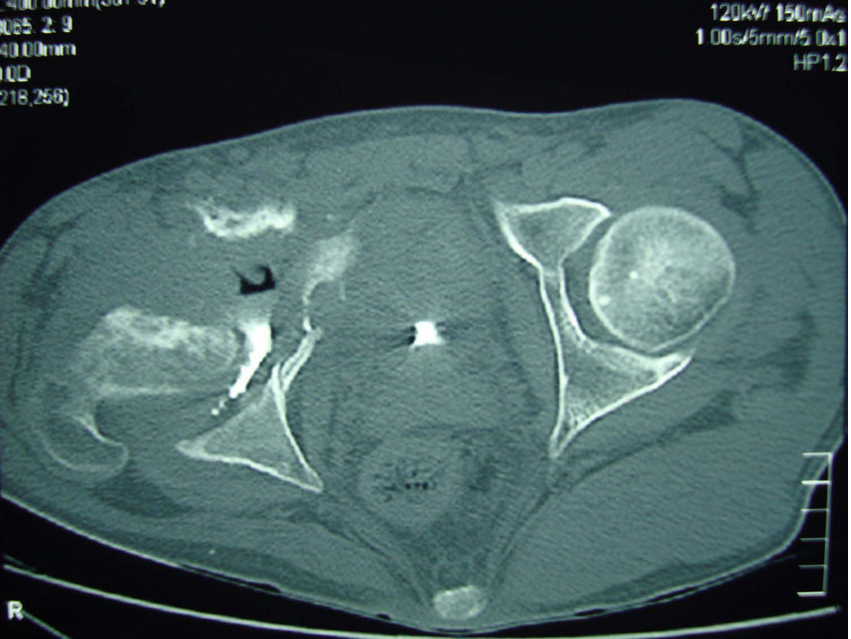
**Axial computed tomography scan of the right acetabular cavity following retrograde urethrography showing destruction of the acetabular cavity and femoral head**. Air-fluid level and contrast media accumulated around the femoral head.

**Figure 3:**
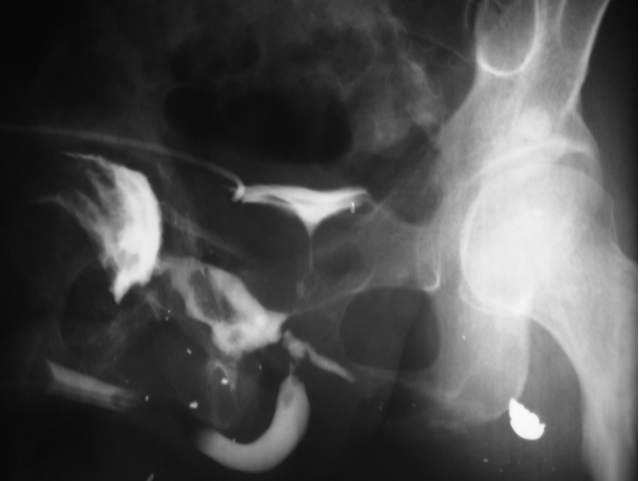
**Left Oblique graphy of the patient's pelvis after retrograde urethrography showing fistula tract and opacification of the right hip joint**.

A 37-year-old Iranian balochi male was shot with a firearm in the upper part of his right pelvis. He underwent primary closure on the same day and a suprapubic cystostomy was placed which was removed 3 weeks later. Ten months later, he developed urinary retention. He underwent retrograde urethrography and antegrade cystography, which showed a stricture measuring 5 cm in length. There was also a history of progressive pain in his right hip joint accompanied by low grade fever which started 2 months after the initial injury. A hip X-ray showed evidence of complicated septic arthritis (Figure 1). There was also accumulation of contrast around the right femoral head and the presence of a fistulous tract between the posterior urethra and his right hip (Figure 2). An axial computed tomography (CT) scan of his pelvis following retrograde urethrography confirmed a fistulous tract with destruction of the acetabular cavity and femoral head (Figure 3). Laboratory tests showed active urinary sediment and positive synovial fluid culture (Table 1). For this, he underwent delayed reconstructive surgery for the urethral stricture using a bladder epithelial graft and urethral fistula via a transperineal approach. The patient was placed in an exaggerated lithotomy position. An inverted Y incision was made in the bulbospongiosus muscle and the muscle displaced laterally. The urethra was released and the edges of the fistula were freshened by passing a curette, followed by a gracilis muscle flap which was placed between the urethra and fistulous tract. The stricture was located by placing a Van Buren sound and semicircular sound in the anterior and posterior urethra via the cystotomy tract, respectively. The stricture length was approximately 5 cm. Stricturectomy was performed and the edges were sutured to a graft from a bladder mucosa. An intraluminal catheter was placed to serve as a stent and a suprapubic cystotomy was done to divert the urine.

**Table 1:** Urine analysis, urine culture, ESR, CBC and hip synovial fluid analysis

Urine analysis	pH 5, SG = 1020, WBC = 8--10, RBC = many, Bacteria = many
Urine culture	colony counts of >10^5^/mL = *Escherichia coli* ESR = 23 mm/hour, 1st hour

CBC	WBC = 12,700 (PMN = 79%, lymphocyte = 20%), RBC = 4.8 × 10^6^, hemoglobin = 11.7, PLT = 237,000

Synovial fluid analysis of hip joint	WBC = 115,000/μL with >90% neutrophils Gram Stain and Culture = *Staphylococcus aureus*

SG, specific gravity; WBC, white blood cell count; RBC, red blood cell count; ESR, erythrocyte sedimentation rate; CBC, complete blood count; PMN, polymorphonuclear neutrophils; PLT, platelets

**Figure 4:**
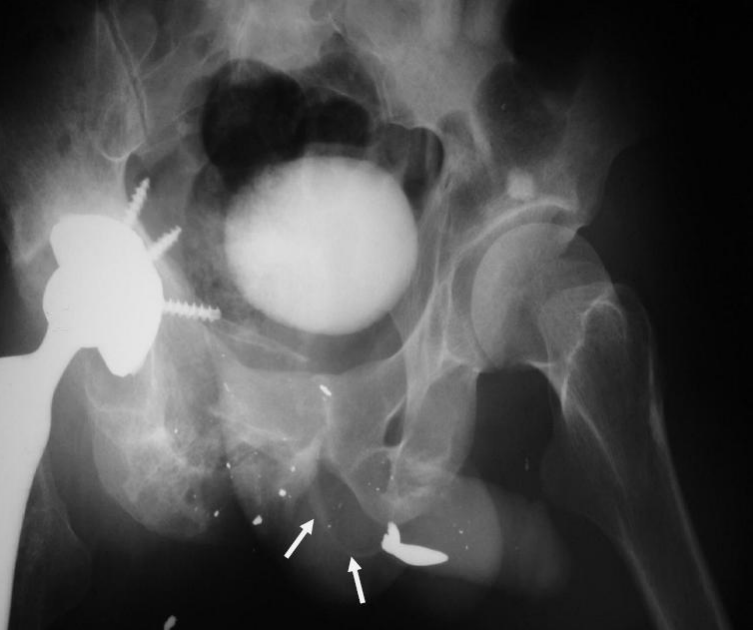
**Left oblique post voiding urethrography of the patient faintly showing patent posterior urethral lumen (arrows)**.

The intraluminal catheter was removed 3 weeks after surgery while the suprapubic cystostomy was clamped and the patient was instructed to void. An antegrade urethrography was performed which showed a widely patent urethra with no evidence of contrast extravasation (Figure 4). After 1 week of normal voiding, the suprapubic catheter was removed. At the same time, the patient was under treatment with antibiotics for his septic arthritis. Four months later (14 months after the initial injury), he underwent non-cemented total hip arthroplasty. The patient was discharged on an antibiotic regimen and followed with post void imaging for 18 months. He was instructed to return for follow-up if he developed difficulty voiding or any reduction in urinary caliber. The patient was not symptomatic during routine follow-up. To our best knowledge, this kind of fistula following a gunshot injury has not been reported previously.

## Discussion

Normally, the urethra may develop an abnormal communication with the bladder, rectum, perineum or genital tract. This communication or fistula can be congenital or acquired. The congenital type is due either to segmental arrest of the embryonic mesoderm that fails to encircle the developing groove at the site of the fistula or to embryonic blowout behind the distal congenital obstruction. The acquired type is mostly due to road traffic accidents or falling from a height.

There is always a possibility of a fistula or sinus tract formation following urethral injury. This can happen following gynecological surgery, obstetric injuries, radiotherapy and some inflammatory conditions (Crohn's disease, peri-urethral abscess, tuberculosis (TB)) or urethral stricture and carcinomas [3,4]. With gynecological surgery, abdominal and vaginal hysterectomies account for almost 75% of reported cases [5]. These fistulas may end in the soft tissue of the perineum or open at the perineal skin or the penis itself. Alternatively, they can end in the rectum, vagina, uterus or the hip. There have also been reports of fistulas ending in the scrotum [6], corpus spongiosum [7] or the corpus cavernosum [8]. The most useful method of direct visualization is fistulography; especially when cutaneous fistulas are concerned [9]. Intravenous urography (IVU) and urethrography are mainstays to diagnose upper urinary tract disorders [9]. Voiding cystourethrography (VCUG) and urethrography are the mainstays to study lower urinary tract symptoms [9]. They show an irregular tract ending in a cavity. Magnetic resonance imaging (MRI) can be helpful to determine the course of the fistula tract and if accompanied by CT, can also show the underlying condition.

A fistulous communication between the posterior urethra and the hip joint is a rare finding. A potentially serious complication of these fistulous tracts is development of septic arthritis of the hip joint. There are five reports of a fistulous tract between the posterior urethra and hip joint following blunt abdominal trauma and all developed septic arthritis [10]. In our patient, there was irreversible hip injury due to delay in diagnosis of the fistula and subsequent septic arthritis. Therefore, early radiologic exclusion of urethral injury via retrograde urethrography and aggressive management by urine drainage using a suprapubic catheter and antibiotic therapy are emphasized to prevent long-term complications such as septic arthritis and destruction of the hip joint [10]. Time of surgical repair is an important factor in the final outcome of the fistula. The surgeon should wait for 3-6 months and, during this period, urine can be diverted by way of a suprapubic cystostomy. There are three main surgical approaches in treatment of fistulas: excision of the tract, freshening of its edges using a surgical technique thereby stimulating scar formation with spontaneous healing, and mobilization of the tissue around the fistula to cover it completely [5,11].

## Conclusion

Although urethral injuries are rare, hip joint contamination with urine following a urethro-acetabular fistula can lead to severe and disabling complications such as septic arthritis. We recommend that every clinician should keep these fistulas in mind as a complication of penetrating urethral injury and every attempt should be made for their early diagnosis and prompt treatment. This case demonstrates that any sign of hip joint involvement in urethral injury, regardless of the cause, needs to be evaluated immediately. The purpose of our report is to emphasize the clinical importance of septic arthritis of the hip following penetrating urethral injuries.

## Abbreviations

VCUG: voiding cystourethrography; CT: computed tomography; MRI: magnetic resonance imaging; TB: tuberculosis; IVU: intravenous urography.

## Consent

Written informed consent was obtained from the patient for publication of this case report and any accompanying images. A copy of the written consent is available for review by the Editor-in-Chief of this journal.

## Competing interests

The authors declare that they have no competing interests.

## Authors' contributions

AR was the radiologist who diagnosed the problem and RH was the assistant in the radiology. BN and AS collected the data and helped draft the manuscript. MG was a major contributor in writing the manuscript. All authors read and approved the final manuscript.
